# Development of a novel risk score reflecting the relative harm potential of synthetic cannabinoids based on prevalence estimates, well‐documented intoxication cases and basic pharmacological data

**DOI:** 10.1111/add.70268

**Published:** 2025-12-21

**Authors:** Michaela J. Sommer, Katharina Elisabeth Grafinger, Maren Hermanns‐Clausen, Volker Auwärter

**Affiliations:** ^1^ Institute of Forensic Medicine, Forensic Toxicology, Medical Center – University of Freiburg Freiburg Germany; ^2^ Hermann Staudinger Graduate School University of Freiburg Freiburg Germany; ^3^ Faculty of Medicine University of Freiburg Freiburg Germany; ^4^ Institute of Forensic Medicine, Forensic Toxicology and Chemistry University of Bern Switzerland; ^5^ Poisons Information Center, Department of General Pediatrics, Adolescent Medicine and Neonatology, Center for Pediatrics, Medical Center – University of Freiburg Freiburg Germany

**Keywords:** [^35^S]‐GTPγS, intoxications, novel psychoactive substances, poison severity score, risk score, synthetic cannabinoid receptor agonists

## Abstract

**Background and aims:**

While the health hazards of synthetic cannabinoid receptor agonists (SCRAs) are often approximated using in vitro pharmacological parameters as surrogate, this approach fails to consider pharmacokinetic and pharmacodynamic complexity. This study aimed to develop a practical risk score for SCRAs prevalent in Germany between 2013 and 2021, based on prevalence data and differentiated data on intoxication cases in the same period of time.

**Design:**

The score integrates data from routine forensic serum/blood and urine analysis and clinical data from a prospective study involving emergency department patients after consumption of new psychoactive substances (NPS) (symptoms and quantification data).

**Setting:**

SCRA prevalence data were obtained from a database query of routine serum/blood and urine analyses conducted at the Institute of Forensic Medicine Freiburg (2013–2021). Intoxication case data were obtained from a prospective study of patients treated in German emergency departments and reported to the Poisons Information Center Freiburg.

**Participants/Cases:**

During the study period, 9929 serum/blood and 45 464 urine samples were routinely analysed for SCRAs. Forty‐eight non‐fatal intoxications with clinical symptoms were included, with written consent provided by participants.

**Measurements:**

Twelve SCRAs were selected based on their prevalence. Quantification in serum/blood and urine samples was conducted with liquid chromatography tandem mass spectrometry. The human cannabinoid receptor 1 affinities and activities were assessed using a competitive radioligand binding assay with [^3^H]CP‐55940 and the functional [^35^S]GTPγS assay.

**Findings:**

From 2013 to 2021, 1633 serum/blood and 8030 urine samples tested positive for one or more SCRAs (positive rate serum/blood: 16.5%; urine: 17.7%). The risk score comprises three parts: (1) SCRA prevalence in routine case samples relative to the occurrence of intoxication cases in a certain time‐frame; (2) the Poison Severity Score; and (3) Toxicological Significance Score, to account for the extent to which each substance contributed to the observed clinical symptoms and consequently to the overall intoxication. The higher the risk score value, the greater the associated toxicological risk of an SCRA in the test set. Risk score values ranged from 9.9 (5F‐PB‐22) to 3.1 (5F‐Cumyl‐PEGACLONE). No inverse relationship was observed between the risk score ranking and the receptor affinity or activity values based on visual comparison of the respective plots.

**Conclusions:**

In vitro potency of synthetic cannabinoid receptor agonists (SCRAs) does not clearly correlate with harm potential, and hence other properties of the SCRAs seem to be involved. The risk score introduced here provides a novel framework for assessing the potential hazards of emerging SCRAs by integrating routine forensic data with clinical intoxication case analyses.

## INTRODUCTION

The rapid emergence of new psychoactive substances (NPS) is reshaping the global drug landscape, challenging scientific, legal and public health communities to keep pace with their evolving complexity [1, 2]. According to the United Nations Office on Drugs and Crime (UNODC), NPS are defined as ‘a new narcotic or psychotropic drugs, in pure form or in preparation, that is not controlled by the 1961 United Nations Single Convention on Narcotic Drugs or the 1971 United Nations Convention on Psychotropic Substances, but which may pose a public health threat comparable to that presented by substances listed in these conventions’ [3]. In Europe, more than 1000 NPS have been monitored since the establishment of the Early Warning System (EWS) in 2005 by the European Union Drug Agency (EUDA) [4, 5]. NPS are classified into different categories based on their chemical structure such as synthetic cannabinoids [also known as synthetic cannabinoid receptor agonists (SCRAs)], synthetic opioids, phenethylamines, piperazines, tryptamines, piperidines and benzodiazepines [6]. SCRAs constitute the largest class of NPS, mimicking the effects of Δ^9^‐tetrahydrocannabinol (THC), the primary psychoactive component of *Cannabis sativa*, by binding to the human cannabinoid receptors subtypes 1 and 2 (hCB_1_ and hCB_2_). The earliest SCRA NPS originated from pharmaceutical research, such as the SCRA JWH‐018 [7] or the tryptamine α‐methyltryptamine (AMT) [8, 9]. However nowadays, the chemical structure of an NPS is modified primarily to circumvent legislation, with little regard for changes in their pharmacological or toxicological properties [8, 10, 11]. Hence, these changes may result in more potent and/or toxic compounds. For SCRAs in particular, a large number of publications on adverse effects, non‐fatal and fatal intoxications can be found in the scientific literature [12, 13, 14, 15, 16, 17, 18, 19].

Standardised scoring systems are used for qualitative assessment and to improve the comparability of poisoning data (e.g. for epidemiological research). To assess the severity of intoxication, poison information centres and clinicians use the Poison Severity Score (PSS), a standardised clinical tool for classifying poisonings in individual patients. The score considers clinical manifestations, the need for medical intervention and the patient's prognosis [20]. In contrast to the patient‐centred PSS, the Toxicological Significance Score (TSS) focuses on assessing the contribution of an NPS to an intoxication event [21]. The TSS was developed to assess and classify the specific role of a certain NPS in fatalities. The TSS is typically determined considering the concentration of the substance, its known toxicological effects and the presence of additional contributing factors (e.g. poly‐drug use). In the present study, it was used to assess the role of a certain SCRA in intoxications. The main difference between PSS and TSS is that in TSS the focus is on the role of a single substance, whereas in PSS the overall clinical picture is decisive.

Because of the dynamic nature of the NPS drug market, it is imperative to pharmacologically and toxicologically characterise these compounds to take appropriate legal and regulatory measures. Currently, in vitro studies are used to pharmacologically characterise the affinity and functional activity of NPS at their respective target receptor [22, 23]. Because of the lack of in vivo data, regulatory risk assessment integrates these pharmacological in vitro data to estimate potential adverse health outcomes for humans (in vivo data). This methodology, however, fails to consider factors such as blood–brain barrier penetration [24], metabolite formation [25] or the broader pharmacological activity of metabolites [26] and differences in down‐stream effects following receptor activation [23], all of which could potentially play a significant role in the overall effects and toxicity of an NPS. In addition, toxic mechanisms mediated by other targets than the typical receptors or transporters could play a role.

**FIGURE 1:**
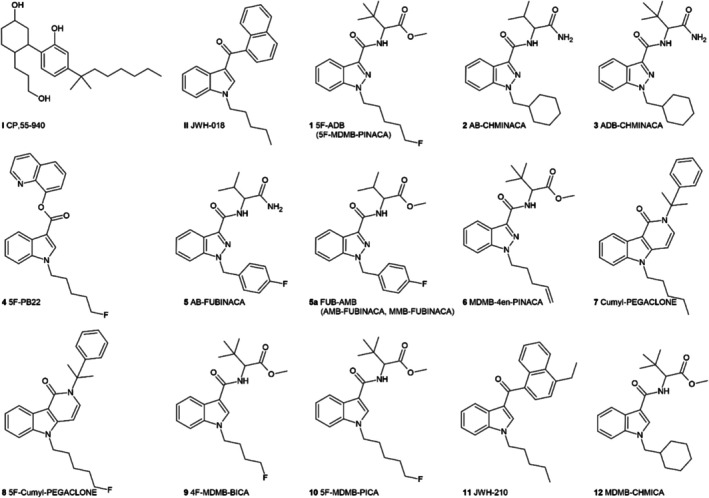
Structures of the 12 investigated synthetic cannabinoid receptor agonists (SCRAs) and the two reference compounds CP‐55940 and JWH‐018.

In this sense, the aim of the present study was the development of a novel risk score for the assessment of 12 different SCRAs (see Figure 1), to achieve better comparability of the health threats and hazards elicited by NPS, insofar as these have not yet been fully characterised toxicologically. Further, the determined scores for the investigated SCRAs are discussed in relation to the pharmacological data available to date (hCB_1_ affinity and activity).

We therefore hypothesise that the actual health risks of an SCRA can be assessed by combining prevalence data (available from routine forensic case work) and data from a prospective clinical study of well‐documented SCRA intoxication cases using a mathematical model that takes into account the long‐established PSS and the TSS.

## METHODS

### Design of the study

To develop a score that allows comparison of the actual health risks of NPS, prevalence data for a compound generated from routine forensic serum/blood and urine analysis were put in relation to a mathematical term representing the frequency of occurrence of a substance in intoxication cases enrolled in a prospective clinical study, resulting in a value greater than 1 if the substance is over‐represented in intoxication cases and a value less than 1 if it is under‐represented. Therefore, higher values indicate substances with a higher risk. The model was established based on the assumption that compounds detected more frequently in intoxication cases than in routine serum/blood or urine samples exhibit higher toxicity and are more likely to cause adverse effects. In contrast, compounds commonly detected in biological samples, but rarely associated with intoxications were considered to be of lower toxicological relevance. To take into account the severity of symptoms and the contribution of the specific NPS, two further summands were introduced. The first is based on the PSS, the second on the TSS. Again, higher values were associated with more severe symptoms and a stronger role of the NPS in causing the symptoms.

The analysis was not pre‐registered on a publicly available platform and the results should be considered exploratory.

### Compound selection

For this study, the 10 SCRAs (AB‐CHMINACA, ADB‐CHMINACA, AB‐FUBINACA, FUB‐AMB, Cumyl‐PEGACLONE, MDMB‐4en‐PINACA, MDMB‐CHMICA, 5F‐MDMB‐PICA, 5F‐ADB and 5F‐PB‐22) which were most frequently detected in intoxications in a prospective observational study during the time period of 2013 to 2021 were selected (see Table 1). Additionally, JWH‐210, a model compound representative of the early SCRAs, along with two substances prevalent in screenings during years 2019/2020 (5F‐Cumyl‐PEGACLONE and 4F‐MDMB‐BICA), were included in this study (see Tables S2–S7). CP‐55940 was selected as the reference compound because of its widespread use as a pharmacological tool in cannabinoid receptor studies [27, 28]. JWH‐018 was also included as a reference compound because of its historical use in this capacity [29, 30]. Including both reference compounds facilitates direct comparison with previously published data, thereby enhancing the relevance and interpretability of the results obtained in the present study [31, 32, 33, 34]. Information on the origin of the test and reference compounds and all chemicals used in this study can be found in Table S1.

**TABLE 1:** Most prevalent SCRAs in 139 intoxication samples included in the prospective study from 2013 to 2021.

SCRA	No. of intoxications	Percentage in %
5F‐ADB	33	24
AB‐CHMINACA	33	24
MDMB‐CHMICA	31	22
ADB‐CHMINACA	18	13
5F‐PB‐22	13	9.4
5F‐MDMB‐PICA	15	11
FUB‐AMB/AB‐FUBINACA^a^	32	23
Cumyl‐PEGACLONE	5	3.6
MDMB‐4en‐PINACA	6	4.3
JWH‐210	3	2.2
4F‐MDMB‐BICA	2	1.4
5F‐Cumyl‐PEGACLONE	1	0.72

*Notes*: Ordered from highest to lowest percentage; percentage refers to proportion of intoxications in which the substance was detected (sum is more than 100% because of multiple SCRAs detected in some cases). Only 48 of these were selected for further evaluation in this study according to the inclusion criteria.

Abbreviation: SCRAs, synthetic cannabinoid receptor agonists.

^a^ AB‐FUBINACA and FUB‐AMB cannot be differentiated in the urine screening because of having the same main metabolite, and therefore, have been evaluated together.

### Routine samples and intoxication cases

Data from routine serum/blood and urine analysis conducted at the Institute of Forensic Medicine Freiburg between January 2013 and December 2021 were used to assess the prevalence of SCRAs. In cases where serum was not available because of haemolysis (e.g. post‐mortem investigations), blood was analysed with the same method. The dataset was obtained through a database query and included cases that met the following inclusion criteria: analytical analysis performed using the selected multiple reaction monitoring (sMRM) method ‘Determination of SCRAs in serum, blood and plasma’ and ‘Quantification of metabolites of SCRAs in urine samples’, which are the standard methods for detection of these substances in our laboratory. The vast majority of serum/blood samples were from forensic medicine institutes or forensic toxicological laboratories in Germany. The urine samples were mostly from prisons, forensic‐psychiatric clinics or from abstinence programs. As the availability of NPS at a certain time can be regarded the same for all subpopulations, a generalisation of the prevalence data gained from these samples seems justified.

The data on intoxication cases used for the development of this risk score were obtained from a prospective study [26]. This study included patients who were treated in an emergency department room following the consumption of an NPS. Clinical symptoms and follow‐up information were documented using a structured questionnaire completed by the attending physicians. The methodology has been previously published [35]. Residual serum (obtained from venous blood) and urine samples, accompanied by the completed questionnaires, were sent to the Poisons Information Center Freiburg for further analysis. The toxicological analyses of serum and urine samples were conducted at the Institute of Forensic Medicine Freiburg. No data was available on previous consumption patterns or potential development of tolerance after continued misuse. The study was conducted in accordance with the Declaration of Helsinki and approved by the regional ethics committee of the University of Freiburg (no. 235/13_130683). The intoxication cases included in this study are those in which SCRA use was confirmed by liquid chromatography tandem mass spectrometry (LC–MS/MS).

### LC–MS/MS analysis of serum/blood and urine samples

Serum/blood and urine analyses were conducted at the Institute of Forensic Medicine Freiburg using previously published LC–MS/MS methods [36, 37], that were modified by adding further analytes present in ‘legal‐high’ products (‘herbal blends’, ‘C‐liquids’ or ‘research chemicals’), and covered approximately 100 different SCRAs. Detailed information can be found in the Supporting Information.

### [^3^H]CP‐55940 in vitro hCB_1_ receptor affinity assay and [^35^S]GTPγS in vitro hCB_1_ functional activation assay

The hCB_1_ receptor affinity and activity of the 12 test compounds were assessed using the competitive [^3^H]CP‐55940 radioligand assay and the functional [^35^S]GTPγS assay, as previously published [22, 38]. Receptor assays were performed on three different days and each concentration was tested in duplicate.

### Data analysis

Raw data were processed using Microsoft Excel, and data were analysed using GraphPad Prism (Version 8.0.2, GraphPad Software).

#### hCB_1_ receptor affinity assay

The maximal inhibitory concentration (IC_50_) values were determined at the turning point of the sigmoidal graph (semi‐logarithmic scale of the horizontal axis), which was generated using One Site‐Fit K_i_ competitive binding function with K_D_ (0.05 nM) specific for hCB_1_ membrane preparations (HEK293‐EBNA, PerkinElmer). From the IC_50_, the respective K_i_ values were calculated using the Cheng–Prusoff equation [39].

#### hCB_1_ receptor activity assay

Curve‐fitting of concentration–response curves was performed via non‐linear regression [dose–response: stimulation; log (agonist) vs. response (three parameters), standard setting: Hill slope = 1; required for the implementation of the intrinsic relative activity model], to determine EC_50_ (a measure of potency) and E_max_ (a measure of efficacy) values. The [^35^S]GTPγS data was normalised to the maximum signal of CP‐55940 (arbitrarily set to 100%), which was used as the reference compound.

## RESULTS AND DISCUSSION

### SCRAs in serum/blood and urine routine samples

Between 2013 and 2021, a total of 9929 serum/blood and 45 464 urine samples were analysed as part of routine testing at the Institute of Forensic Medicine Freiburg. Of these, 1633 serum/blood and 8030 urine samples were positive for one or more SCRA.

**FIGURE 2:**
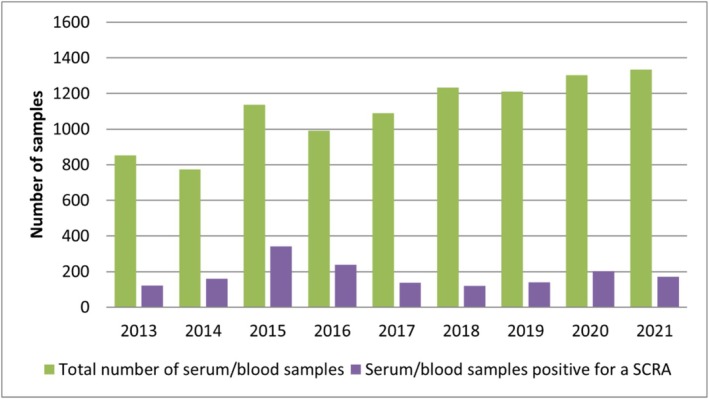
Annual total number of analysed serum/blood samples (green) compared to the number of samples containing at least one synthetic cannabinoid receptor (SCRA) (purple); study period: 2013–2021.

**FIGURE 3:**
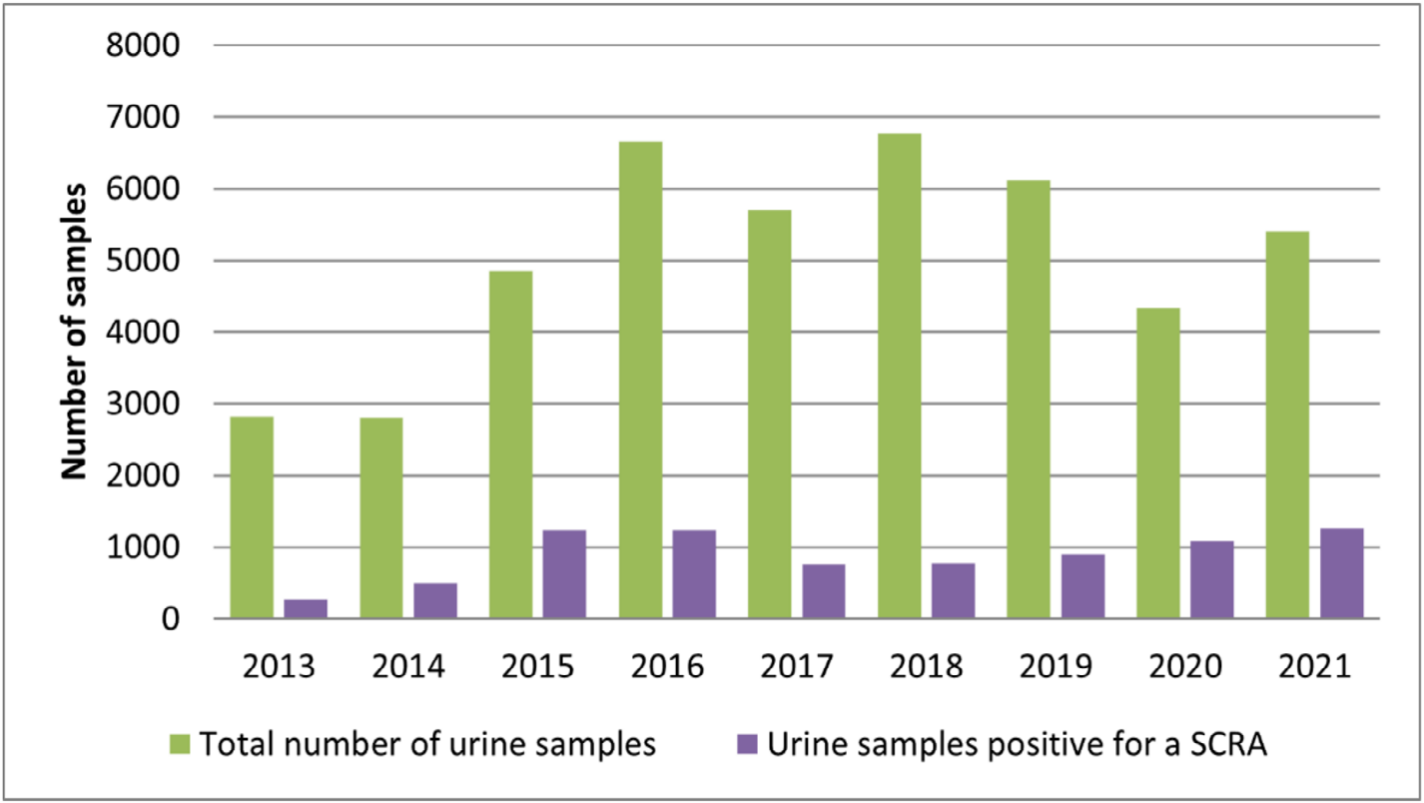
Annual total number of analysed urine samples (green) versus the number of samples positive for at least one synthetic cannabinoid receptor (SCRA) (purple); study period: 2013–2021.

The proportion of SCRA‐positive serum/blood samples ranged from 7% (fourth quarter 2018) to 38% (first quarter 2015). Similarly, the proportion of SCRA‐positive urine samples ranged from 7% (fourth quarter 2013) to 30% (fourth quarter 2020). The general trend of positive serum/blood and urine samples was similar over the years (see Figures 2 and 3). For more details on the results of the analysed routine forensic case samples see Tables S4–S7.

### Determination of PSS (G_2_) and TSS (G_3_)

To determine PSS and TSS, the clinical symptoms of SCRA intoxications were used alongside the toxicological results of the analysis of urine and serum samples from a prospective study involving patients who were treated in emergency departments after consuming an NPS. Forty‐eight cases were included in the present study [26]. In Table S8, detailed quantification results and recorded symptoms are listed for two examples. For details on the respective SCRA statistics per quarter in the analysed intoxication case samples see Table S2–S3.

The recorded symptoms were usually non‐specific and could range from mild (e.g. headaches) to life‐threating (e.g. respiratory insufficiency or extreme agitation). The PSS was determined based on the described symptoms. Comparing two cases of AB‐CHMINACA intoxication (case A and B) provides a better understanding of how the PSS and TSS were determined. In case A, the presence of severe bradycardia (35 bpm/minutes) alone was sufficient to categorise the case as a severe poisoning (PSS grade 3). Agitation and seizures further supported systemic toxicity. In case B, although the presence of disorientation and miosis suggests significant central nervous effects, these do not indicate life‐threatening complications such as repeated seizures, status epilepticus or respiratory failure. Furthermore, disorientation and restlessness are commonly experienced during synthetic cannabinoid intoxication, which reinforces the classification as moderate (PSS grade 2).

When evaluating the TSS, the serum concentrations of AB‐CHMINACA and other drugs of abuse were considered. In case A, an AB‐CHMINACA concentration of 9.5 ng/mL was determined. Additionally, a concentration of 320 ng/mL of the synthetic cathinone 3‐MMC was found in the serum sample. Therefore, the contribution of AB‐CHMINACA to the intoxication was categorised as ‘mild’ (TSS grade 2). However, in case B, an almost three times higher AB‐CHMINACA concentration of 27 ng/mL was determined in the serum. The other four confirmed SCRAs were only present at very low concentrations. Hence, the contribution of AB‐CHMINACA to the intoxication was categorised as ‘dominant’ (TSS grade 3) in this case.

### Development of a risk score for the assessment of health risks associated with SCRAs

For the purpose of risk assessment of a SCRA, several statistical evaluations were integrated into a descriptive composite measure, consisting of three pre‐defined components, termed the risk score. Its development is based on prevalence data from routine forensic case work and intoxication cases with analytical measurements and clinical symptoms.

The relationship between the prevalence of a SCRA in serum/blood and urine samples and its prevalence in intoxication cases is fundamental to the development of the risk score. This approach hypothesises that substances that are frequently detected in biological samples, but rarely associated with intoxication cases are likely to be less toxic or hazardous compared to those that are less commonly detected, but frequently associated with intoxications in the present study. A significant challenge, however, is the dynamic nature of the NPS market [41], leading to varying consumption time‐frames for each compound. Additionally, because many cases included in this study were poly‐intoxications, the risk score must consider the extent to which each substance contributed to the clinical symptoms and overall intoxication. Hence, the severity of intoxication (PSS, G_2_) [20], as well as the influence of the SCRA on the intoxication (TSS, G_3_) [21] was considered in the novel risk score.

The risk score is composed of three components (parts G_1_, G_2_ and G_3_) and was designed as follows:

(1) $$G\left(x\right)=G_{1}\left(x\right)+G_{2}\left(x\right)+G_{3}\left(x\right),$$

The initial considerations for part 1 of the risk score resulted in equation 2. Equation 2 takes into consideration several components: the number of intoxications with SCRA *x* in quarter *k* over the total number of intoxications of all investigated SCRAs in the same quarter; the number of serum/blood samples positive for SCRA *x* over the total number of serum/blood samples positive for all SCRAs plus the number of urine samples positive for SCRA *x* over the total number of urine samples positive for all SCRAs (this is divided by two to account for the two different sample types); and the number of quarters in which the SCRA *x* was prevalent.

(2) G1x=∑k=1gakbkckdk+ekfk2h+i

*a*_*k*_: number of intoxications with the SCRA *x* in quarter *k*;
*b*_*k*_: total number of intoxications with any SCRA in quarter *k*;
*c*_*k*_: number of serum/blood samples tested positive for SCRA *x* in quarter *k*;
*d*_*k*_: total number of serum/blood samples tested positive for any SCRA in quarter *k*;
*e*_*k*_: number of urine samples tested positive for SCRA *x* in quarter *k*;
*f*_*k*_: total number of urine samples tested positive for any SCRA in quarter *k*;
*g*: total number of all quarters;
*h*: number of quarters in which the SCRA *x* was present in serum/blood or urine samples: cd+ef2≥0.05; and
*i*: number of quarters in which at least one intoxication occurred, but cd+ef2<0.05 (the prevalence of the SCRA *x* in serum/blood and urine samples is lower than 5% on average).

(3) G1x=∑k=1g2·abcd+ef*h+i−1

Consequently, equation 2 was mathematically rearranged. To further simplify the equation and reduce the formula signs, in equation 3 the following simplifications were taken: $\frac{a}{b\;}=a_{i}$, $\frac{c}{d\;}=a_{s}$ and $\frac{e}{f\;}=a_{u}$. This results in the final equation 4 for G_1_:

(4) $$G_{1}\left(x\right)=\frac{2}{q_{p}+q_{i}}\cdot \sum_{k=1}^{n}{\left(\frac{a_{i}}{a_{s}+a_{u}}\right)}_{k};$$

*a*_*i*_ *:* proportion of intoxications with the SCRA *x* quarter *k*;
*a*_*s*_ *:* proportion of serum/blood samples positive for SCRA *x* in the quarter *k*;
*a*_*u*_ *:* proportion of urine samples positive for SCRA *x* in the quarter *k*;
*q*_*p*_ *:* number of quarters in which the SCRA *x* was present in urine or serum samples, as+au2≥0.05;
*q*_*i*_ *:* number of quarters in which at least one intoxication with SCRA *x* occurred, but as+au2<0.05; and
*n:* number of all quarters.

Hence, to calculate $G_{1}\left(x\right)$, the quotient of the proportion of intoxications with substance *x* in all NPS intoxications, and the average proportion of positive serum/blood and urine samples with substance *x* were calculated for each quarter. The sum of these quotients was divided by the number of all prevalent quarters (*q*_*p*_). Prevalent quarters were defined as quarters in which on average the substance was detected in more than 5% of the positive serum and urine samples. If a poisoning occurred in a quarter in which the substance was not prevalent, the number of these quarters was added (*q*_*i*_). This part of the score has similarities with an OR [42]. If the proportion of routine samples in a given quarter in which an intoxication with the respective substance occurred was zero (*a*_*s*_ + *a*_*u*_ = 0), a value of 0.01 was used for that quarter in the calculation of the quotient to avoid division by zero.

For part G_2_, the severity of the intoxication caused by the respective SCRA *x* was assessed on the basis of reported symptoms and follow‐up information using the PSS [20]. The PSS classifies the intoxication severity into five levels: (0) no, (1) mild, (2) moderate, (3) severe, and (4) fatal intoxication. The most severe reported clinical symptom determines the severity of the intoxication, hence the score. For this purpose, the mean value of the PSS of all intoxications with the respective SCRA *x* was calculated ( $G_{2}\left(x\right))$.

Because the present study included only non‐fatal intoxications with PSS values of 1, 2 or 3, the values calculated for $G_{2}\left(x\right)$ for each compound ranged from 1.6 to 2.2. A value closer to 1 indicates that the respective SCRA was detected primarily in cases with mild intoxication symptoms, while a value closer to 3 corresponds to SCRAs associated with more severe intoxication symptoms.

For part G_3_, the involvement of the SCRA in the respective intoxications was assessed. This was evaluated on the basis of the TSS [21]. Elliott *et al*. [21] developed the TSS to assess and classify the contribution of NPS in fatalities. In the present study, the TSS was determined to weigh the involvement of the investigated SCRA in the intoxication and the contribution of the SCRA to the observed symptoms. To determine the TSS value, factors such as the determined concentration of the SCRA, the presence of other recreational drugs or medicinal drugs, the general health of the patient and the consumption circumstances need to be considered. A TSS value of 1 indicates that another compound or other factors were responsible for the intoxication (Table 2). A value of 2 was assigned to cases where the substance may have contributed, but other compounds (recreational or medicinal drugs) with potentially higher toxicological significance were present. In contrast, a TSS value of 3 indicates that the SCRA was mainly responsible for the intoxication, even in the presence of other drugs. In cases with more than one drug detected, a TSS of 3 was only assigned where other compounds were present at concentrations below the range in which relevant effects are to be expected.

**TABLE 2:** TSS scoring and classification taken from Elliott *et al*. [21].

Score value	Level of significance	Elucidation
1	Low	Alternative cause of death
2	Medium	NPS may have contributed to toxicity/death, other drugs present may be more toxicologically significant
3	High	NPS cited as cause of death or has been cited as likely to have contributed to toxicity/death (even in the presence of other drugs)
U	Unclassified	Insufficient data to allow assessment

Abbreviations: NPS, new psychoactive substances; TSS, Toxicological Significance Score.

For the risk score, the mean value of the TSS of all intoxications with substance *x* was calculated ( $G_{3}\left(x\right))$. Analogous to the value of $G_{2}\left(x\right)$, the value of $G_{3}\left(x\right)$ can theoretically be between 1 and 3. With a value of ‘1’, substance *x* was not mainly responsible for the symptoms in all of the intoxications (low level of significance), and with a value of ‘3’, it was the main cause in every poisoning in which it was detected.

### Results and evaluation of the risk score for the assessment of SCRAs

The resulting risk score (G_total_) and the parts it consists of (G_1_–G_3_) are presented in Table 3, ranked from highest to lowest risk score. The values for G_2_ and G_3_ represent the PSS and TSS, respectively, and are absolute values. Hence, they can theoretically range from 1 to 3.

**TABLE 3:** The calculated risk score (G_total_) and the three parts it consists of (G_1_–G_3_) for the 12 investigated SCRAs.

Ranking		G_1_	G_2_	G_3_	G total
1	5F‐PB‐22	5.3	1.9	2.7	9.9
2	5F‐MDMB‐PICA	3.2	2.1	2.8	8.1
3	AB‐CHMINACA	2.8	2.1	2.3	7.2
4	ADB‐CHMINACA	2.3	2.1	2.7	7.1
5	5F‐ADB	2.5	1.9	2.6	6.9
6	MDMB‐4en‐PINACA	1.6	2.2	3.0	6.8
7	AB‐FUBINACA/FUB‐AMB^a^	1.9	2.0	2.2	6.1
8	JWH‐210	1.4	1.6	2.7	5.7
9	MDMB‐CHMICA	0.73	2.2	2.8	5.7
10	4F‐MDMB‐BICA	0.48	2.0	3.0	5.5
11	Cumyl‐PEGACLONE	0.32	2.0	2.2	4.5
12	5F‐Cumyl‐PEGACLONE	0.11	2.0	1.0	3.1

*Notes*:The compounds are ordered from highest to lowest risk score.

Abbreviation: SCRAs, synthetic cannabinoid receptor agonists.

^a^ AB‐FUBINACA and FUB‐AMB cannot be differentiated in the urine screening because of having the same main metabolite, and hence have been evaluated together.

The following considerations were taken for G_1_. G_1_ has an expected value of 1, if the number of occurrences of a SCRA in serum/blood or urine samples (on average) and in intoxication cases is comparable. G_1_ values greater than 1 result when the SCRA occurs more frequently in intoxication cases than in serum/blood or urine samples. This part of the risk score is based on the assumption that substances with a G_1_ value greater than 1 are to be classified as relatively dangerous and substances with a G_1_ value less than 1 are less likely to cause non‐fatal or fatal intoxications and are therefore considered to be less dangerous.

**FIGURE 4:**
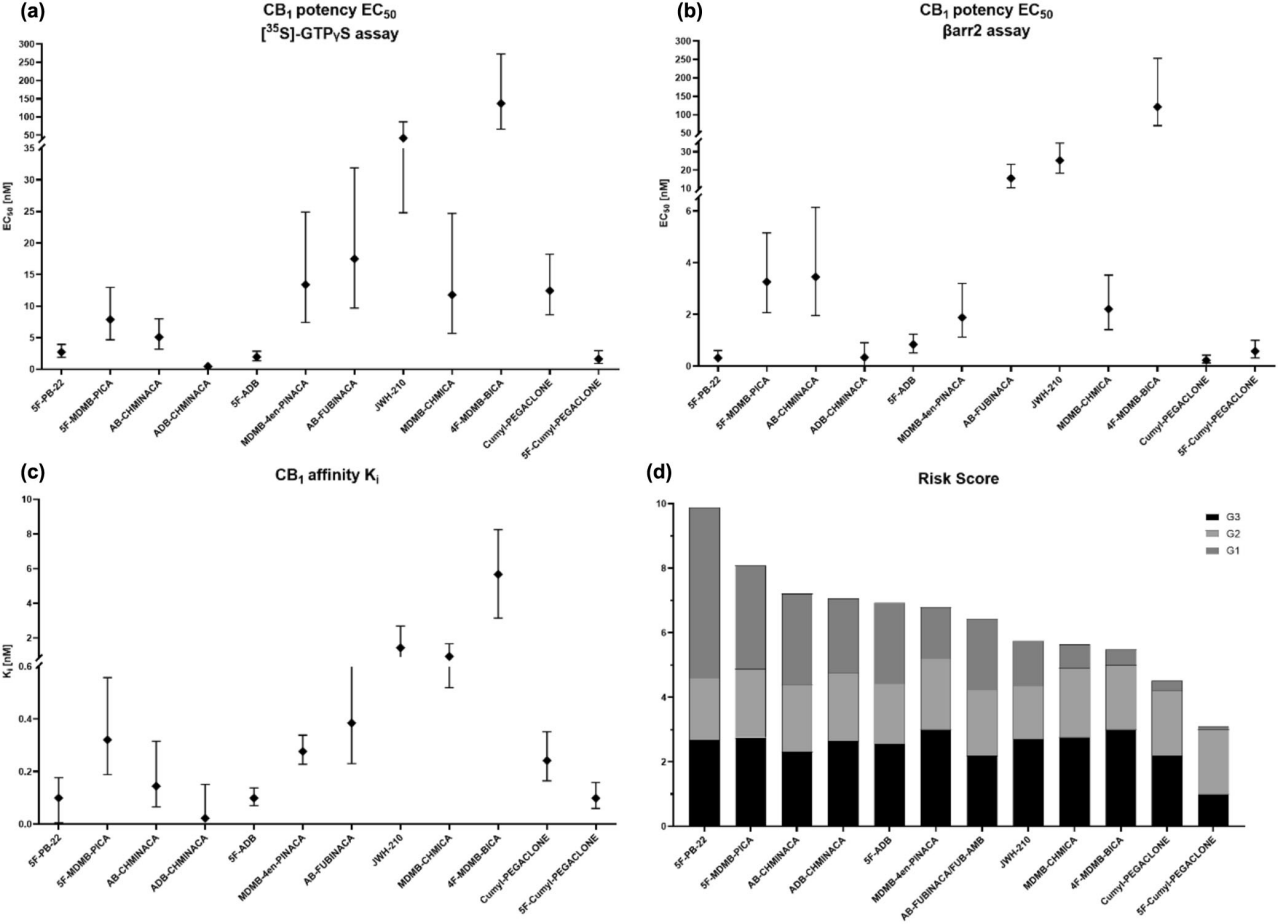
Comparison of the potencies and the risk score for each synthetic cannabinoid receptor (SCRA). Compounds are listed in the order of the risk score (highest to lowest). (a) EC_50_ values per SCRA derived from the [^35^S]‐GTPγS assay; (b) EC_50_ values per SCRA derived from the β‐arrestin2 (βarr2) assay; (c) K_i_ values per SCRA; and (d) risk score for all investigated SCRAs.

In the present study, a G_1_ value lower than 1 was observed for four substances and a G_1_ value higher than 1 was observed for eight substances. Opposed to G_2_ and G_3_, which can only have values between 1 and 3, the value of G_1_ is not capped, and therefore, has a greater influence on G_total_. This is desirable because the G_1_ takes into account the prevalence of a compound in routine forensic case samples as well as the number of intoxication cases in the different quarters (temporal correction). In addition, most G_2_ values are close to 2, hence, G_2_ has the least influence on G_total_ in our study [Figure 4(d) and Table 3].

According to the newly developed risk score, the two most harmful SCRAs were 5F‐PB‐22 and 5F‐MDMB‐PICA (Table 3). These compounds were comparatively frequently identified in those quarters, in which they were under‐represented in routine serum/blood and urine samples (<5% of the positive samples). Substances, which had a low prevalence in routine samples, but were present in intoxication cases, had a high G_1_ score because the ratio $\left(\frac{a_{i}}{a_{s}+a_{u}}\right)$ increased in the respective quarter. Therefore, substances that were present in intoxication cases, but had a low prevalence in routine samples, depicted a high G_1_ score.

The three substances with the lowest G_1_ score were Cumyl‐PEGACLONE, 5F‐Cumyl‐PEGACLONE and 4F‐MDMB‐BICA. In the prospective study, very few intoxications were reported with any of these SCRAs. However, the prevalence of these compounds was rather high in certain quarters for routine analysis samples. Therefore, a rather low G_1_ score was calculated for these compounds.

### hCB_1_ structure activity relationship

The hCB_1_ agonist affinities and activities of the 12 investigated SCRAs were determined using a competitive radioligand binding assay with [^3^H]CP‐55940 and the functional [^35^S]GTPγS assay. The hCB_1_ binding affinities (K_i_ values) are summarised in Table 4 and their concentration‐displacement curves are shown in Figure S1. Table 5 presents the potencies, expressed as EC_50_ values and efficacies represented as E_max_ values relative to CP‐55940 obtained from the functional [^35^S]GTPγS assay. Additionally, for comparison, available literature data from the β‐arrestin2 (βarr2) recruitment assay are depicted in Table 4 (E_max_ data normalised to CP‐55940). The full concentration‐response curves are shown in Figure S2.

**TABLE 4:** Binding affinity parameters of all 12 investigated SCRAs and the two reference compounds at the human hCB_1_ receptor.

No.	SCRA synonym	K_i_ [nM] (95% CI)
I	CP‐55940	1.23 (0.740–1.99)
II	JWH‐018	2.86 (1.77–4.63)
1	5F‐ADB	0.0993 (0.0708–0.138)
2	AB‐CHMINACA	0.145 (0.0661–0.316)
3	ADB‐CHMINACA	0.0231 (0.0150–0.0356)
4	5F‐PB‐22	0.0996 (0.0551–0.177)
5	AB‐FUBINACA	0.385 (0.231–0.662)
5a	FUB‐AMB^a^	10.04 (7.22–14.0
6	MDMB‐4en‐PINACA [38]	0.277 (0.339–0.228)
7	Cumyl‐PEGACLONE	0.242 (0.165–0.352)
8	5F‐Cumyl‐PEGACLONE	0.0991 (0.0603–0.159)
9	4F‐MDMB‐BICA	5.67 (3.15–8.25)
10	5F‐MDMB‐PICA	0.321 (0.189–0.558)
11	JWH‐210	1.426 (0.716–2.69)
12	MDMB‐CHMICA	0.935 (0.520–1.66)

*Notes*: Data are given as K_i_ (nM) both in a 95% CI.

Abbreviations: hCB_1_, human cannabinoid 1; SCRAs, synthetic cannabinoid receptor agonists.

^a^ Data taken from Gamage *et al*. [40].

**TABLE 5:** The receptor activation determined using the [^35^S]‐GTPγS assay in comparison to literature data from the βarr2 assay.

No.	SCRA synonym	[^35^S]‐GTPγS assay		βarr2 assay	
		EC_50_ [nM] (95% CI)	E_max_ [%] (95% CI)	EC_50_ [nM] (95% CI)	E_max_ [%] (95% CI)
I	CP‐55940	24.1 (13.3–44.7)	96.9 (90–104)	0.962^b^ (0.727–1.27)	100^b^ (96.2–104)
II	JWH‐018	38.6 (22.8–64.0)	118 (109–128)	17.8^b^ (10.1–32.7)	306^b^ (281–333)
1	5F‐ADB	1.95 (1.35–2.86)	103 (98.3–108)	0.84^c^ (0.52–1.24)	976.1^a^ (1197–1083)
2	AB‐CHMINACA	5.09 (3.18–7.99)	124 (116–136)	3.45^c^ (1.96–6.14)	1195^a^ (1097–1331)
3	ADB‐CHMINACA	0.467 (0.340–0.638)	124 (119–129)	0.34^c^ (0.02–0.91)	803.6^a^ (724.3–921.4)
4	5F‐PB‐22	2.73 (1.90–3.93)	124 (119–130)	0.32^d^ (0.16–0.61)	980.2^d^ (888.2–1072)
5	AB‐FUBINACA	17.5 (9.69–31.9)	114 (105–123)	15.6^f^ (10.4–23.2)	990.5 (922.9–1059)^a^
	FUB‐AMB	0.543^e^ (0.31–0.95)	69.42 (62.6–76.2)		
6	MDMB‐4en‐PINACA	13.4 (7.44–24.9)	114 (105–124)	1.88^b^ (1.13–3.20)	679 (623–730)
7	Cumyl‐PEGACLONE	12.45 (8.63–18.2)	117 (111–123)	0.23^g^ (0.13–0.43)	1053 (960.8–1148)^a^
8	5F‐Cumyl‐PEGACLONE	1.64 (0.940–2.94)	105 (97.0–113)	0.58^g^ (0.32–1.00)	1089 (988.4–1190)^a^
9	4F‐MDMB‐BICA	137 (66.5–273)	104 (92.7–116)	121^h^ (69.9–253)	774.2 (685.4–911.9)^a^
10	5F‐MDMB‐PICA	7.88 (4.67–13.0)	112 (104–120)	3.26^f^ (2.07–5.15)	1014 (951.7–1075)^a^
11	JWH‐210	41.9 (24.8–86.5)	93.4 (86.5–101)	25.3^i^ (18.3–34.7)	714.2 (672.3–759.5)^a^ ^,^ ^i^
12	MDMB‐CHMICA	11.8 (5.68–24.7)	103 (92.9–114)	2.2^d^ (1.41–3.52)	885.7 (818.5–958.0)

*Notes*: Data is presented as EC_50_ (nM) and E_max_ (%) values in a 95% CI as a measure of potency or efficacy, respectively. The E_max_ is expressed relative to CP‐55940.

Abbreviations: βarr2, β‐arrestin2; SCRAs, synthetic cannabinoid receptor agonists.

^a^ The E_max_ was normalised to JWH‐018 in the original publication, but was here corrected using a correction factor based on the ratio of E_max‐CP‐55940_/E_max‐JWH‐018_ (0.3268), which is found to be consistent in the βarr2 assay (data not shown).

^b^ Data derived from Grafinger *et al*. [27].

^c^ Data derived from Wouters *et al*. [29].

^d^ Data derived from Wouters *et al*. [31].

^e^ Data derived from Gamage *et al*. [40].

^f^ Data derived from Noble *et al*. [30].

^g^ Data derived from Janssens *et al*. [43].

^h^ Data derived from Cannaert *et al*. [44].

^i^ Data derived from Cannaert *et al*. [45].

All 12 investigated compounds were nanomolar to submicromolar CB_1_ ligands (K_i_ = 0.0231–5.67 nM), with only the reference compounds JWH‐018 (K_i_ = 2.86 nM) and 4F‐MDMB‐BICA (K_i_ = 5.67 nM) showing higher CB_1_ affinity than the reference compound CP‐55940 (K_i_ = 1.23 nM). Of the 12 test compounds, ADB‐CHMINACA (K_i_ = 0.0229 nM) presented the highest CB_1_ affinity, followed by 5F‐ADB (K_i_ = 0.0993 nM) and 5F‐PB‐22 (K_i_ = 0.0996 nM). The rank order of potencies observed in the [^35^S]‐GTPγS assay was largely consistent with literature data from the βarr2 assay and comparable to the rank order of hCB_1_ affinities.

For the [^35^S]‐GTPγS the four most potent compounds were ADB‐CHMINACA (EC_50_ = 0.467 nM), followed by 5F‐Cumyl‐ PEGACLONE (EC_50_ = 1.64 nM), 5F‐ADB (EC_50_ = 1.95 nM) and 5F‐PB‐22 (EC_50_ = 2.73 nM), which had potencies within the same CI. Whereas, the four most potent compounds in the βarr2 assay were Cumyl‐PEGACLONE (EC_50_ = 0.23 nM), 5F‐PB‐22 (EC_50_ = 0.32 nM), ADB‐CHMINACA (EC_50_ = 0.34 nM) and 5F‐Cumyl‐PEGACLONE (EC_50_ = 0.58 nM), which were all in the subnanomolar range and had overlapping CIs. In both assays AB‐FUBINACA, JWH‐210 and 4F‐MDMB‐BICA were the least potent compounds.

We were able to show in previous research investigating 30 different SCRAs with three different receptor activity assays ([^35^S]‐GTPγS assay, βarr2 live cell‐based nano‐luciferase assay and mini Gαi live cell‐based nano‐luciferase assay) that EC_50_ values are comparable between assays. However, E_max_ is highly assay‐dependent. Against this background and in the knowledge that the [^35^S]‐GTPγS assay in particular is subject to a ceiling effect [27], this parameter was not considered for the comparison. For the sake of completeness, E_max_ values are nevertheless given in the results tables (Table 5).

### Linking the risk score with pharmacological data

Given that the standard NPS risk assessment approach mostly relies on in vitro pharmacological data, we aimed to compare the results of the developed risk score with the corresponding affinity and activity data of the investigated SCRAs.

To facilitate visualisation and comparison of the data from the three in vitro assays with the risk score, the three pharmacological entities (K_i_, EC_50_ [^35^S]‐GTPγS and EC_50_ βarr2) and the risk factor were plotted for each investigated SCRA. The compounds are arranged in descending order of risk score (highest to lowest, left to right), as shown in Figure 4. It was hypothesised, that higher risk score values would correspond to lower K_i_ and EC_50_ values, reflecting higher receptor affinity and potency, respectively.

Although 5F‐PB‐22 had the highest risk score with a value of 9.9, its hCB_1_ affinity and activity were the fourth highest out of the investigated compounds. Further, 5F‐MDMB‐PICA and AB‐CHMINACA with the second and third highest risk scores, showed even lower ranked hCB_1_ affinities and activities. Similarly, at the lower end of the risk score, represented by 4F‐MDMB‐BICA and 5F‐Cumyl‐PEGACLONE, no clear correlation between the risk score and the pharmacological parameters can be observed. This is evident from their EC_50_ values, with 4F‐MDMB‐BICA exhibiting a value of 137 nM and 5F‐Cumyl‐PEGACLONE showing a significantly lower value of 1.64 nM. Generally, compounds with risk scores in the lowest and highest regions show very similar EC_50_ and K_i_ values. In contrast, there are significant variations in EC_50_ and K_i_ values within the middle range of risk scores, despite the fact that the risk scores are very similar. In summary, no clear relationship was observed between the risk score and the receptor affinity or activity values on the basis of visual comparison.

Interestingly, Cumyl‐PEGACLONE and 5F‐Cumyl‐PEGACLONE, which have potencies in the nanomolar range (EC_50_ = 12.45 nM and 1.64 nM), had the lowest risk scores out of the 12 test compounds. Although both Cumyl‐SCRAs were frequently detected in marketed products (Figure S3) not many intoxications were reported for them, resulting in a very low G_1_ value (compare Figure 4 and Table 3). Based on these findings, both compounds appear to exhibit lower toxicity despite demonstrating relatively high hCB_1_ receptor potencies. Multiple factors may contribute to this effect. Drug producers might be aware of the potencies of certain SCRAs and, consequently, may produce incense blends with lower concentrations to reduce the risk of acute toxic effects. In alignment with this theory, it is plausible that consumers may also be aware of the high potencies of these compounds and consequently adjust their consumed doses to the lower range. Other reasons for the lower risk scores could be differences in pharmacological properties other than potency as assessed by in vitro, receptor‐based activity assays such as blood–brain barrier permeability, pharmacokinetics or off‐target effects attributed to the substances with higher risk scores.

### Limitations of this study

When applying the risk score and interpreting the results, several potential limitations should be considered. First, the numerical value derived from the calculation of G_1_, G_2_ and G_3_ holds limited standalone significance and must always be evaluated in the context of the results for all substances tested within the study period.

The averaged G_2_ values (PSS) for each SCRA were consistently in the range of 2 (PSS scale from 1 to 3), resulting in minimal impact on the overall score. In the present study, the scoring system of the PSS was adopted. To enhance the discriminatory power of G_2_, a subdivided scale with half numbers could be considered. This adjustment would allow for greater variability in scores and facilitate a more nuanced assessment of factors such as mono‐ versus poly‐intoxications, SCRA concentrations, the impact of other consumed drugs of abuse and time since consumption. It has to be noted that the weighting of G_1_, G_2_ and G_3_ in our study was rather arbitrary and may be adjusted in future studies, although we believe that giving G_1_ a higher relative weight seems plausible.

Another limitation is the limited number of intoxication cases available for this study. Accordingly, cases in which drugs other than SCRAs were detected were also included in this study. Although data for some substances encompassed up to 30 intoxication cases and included mono‐intoxications, novel compounds such as 5F‐Cumyl‐PEGACLONE and 4F‐MDMB‐BICA were documented in only a few intoxication cases. This discrepancy may reflect the compounds prevalence during the study period, since they were more frequently detected in later years. Alternatively it may indicate that their consumptions may result in fewer non‐fatal intoxications.

In general, consumer behaviour appears to have shifted during the study period. On the one hand, SCRA producers possibly showed higher awareness of the high hCB_1_ affinity and activity of novel compounds and may, therefore, have shifted production toward herbal blends with lower SCRA concentrations. On the other hand, consumers may have become more conscious of the associated health risks, leading to more caution when consuming these compounds. Moreover, new products such as vaping liquids have become more popular.

A further limitation is that the components of the risk score are themselves estimates and, therefore, subject to uncertainty. Future studies should explore approaches to account for this more explicitly.

Finally, another limitation of the present study is that the risk score assumes that the prevalence of compounds in serum/blood or urine samples can reflect general exposure and toxic potential. However, measured concentrations are influenced by several pharmacokinetic and pharmacodynamic factors, including the time interval between consumption and sampling, compound potency, metabolism and user tolerance. Highly potent SCRAs may cause severe toxicity at low concentrations, while chronic users may exhibit elevated serum levels without corresponding clinical severity. Furthermore—although SCRA metabolites seem to be mostly pharmacologically inactive [45]—potentially active metabolites were not considered in the current analysis. Future studies should aim to incorporate these factors to refine the interpretation of compound prevalence and associated risks.

## CONCLUSION

The present study introduces a novel risk score for the assessment of SCRAs based on prevalence data from routine forensic analyses and intoxication cases. This study included 12 different SCRAs that showed a high prevalence in Germany between 2013 and 2021.

Traditionally, the potential risk of novel SCRAs is assessed by their pharmacological characterisation, mainly via in vitro hCB_1_ affinity and activity measurements. Our findings show that such in vitro data cannot be directly extrapolated to estimate the relative harm, as indicated by the comparison of the risk score data and pharmacological data (hCB_1_ affinity and activity). Hence, other properties of the SCRAs such as blood–brain barrier permeability, pharmacokinetics or off‐target effects seem to play a role and need further evaluation.

In the present study, we demonstrated how the developed risk score offers a novel approach and an alternative method to assess the hazard and toxicity of NPS.

## AUTHOR CONTRIBUTIONS

**Michaela J. Sommer**: Writing—original draft (equal); conceptualization (equal); validation (equal); methodology (equal); investigation (equal); formal analysis (equal); data curation (equal); visualization (equal). **Katharina Elisabeth Grafinger**: Writing—original draft (equal); conceptualization (equal); methodology (equal); investigation (equal); formal analysis (equal); data curation (equal). **Maren Hermanns‐Clausen**: Writing—review and editing (equal); investigation (equal); data curation (equal). **Volker Auwärter**: Writing—review and editing (equal); conceptualization (equal); methodology (equal); supervision (equal).

## DECLARATION OF INTERESTS

None

## Supporting information


**Figure S1:** Percentage of the respective SCRAs included in this study in herbal incense samples, serum/blood and urine samples analysed in the Institute of Forensic Medicine Freiburg in the study period 2013 to 2021.
**Figure S2:** Concentration‐displacement curves at the human CB_1_ receptor derived from the competitive [^3^H]CP,55940 mediated in vitro receptor affinity assay upon the concentration‐dependent stimulation with the twelve test compounds and two reference compounds. Data given as mean receptor affinity ± SEM (n = 3 or higher).
**Figure S3:** Concentration dependent interaction of [^35^S]‐GTPγS with the human CB1 upon stimulation with the twelve test compounds. The data is depicted as mean receptor activation ± SEM (n = 3), normalised to the E_max_ of CP‐55940 (100 %).
**Table S1:** Materials.
**Table S2:** Number of total and individual SCRA intoxications per quarter in the study period from 2013 to 2021.
**Table S3:** Percentage of individual SCRA intoxications in the respective quarter in the study period from 2013 to 2021.
**Table S4:** Total number of analyzed serum/blood samples for SCRAs per quarter in the study period from 2013 to 2021. Number of serum/blood samples positive for at least one SCRA and serum/blood samples positive for each individual SCRA.
**Table S5:** Percentage of serum/blood samples positive for each individual SCRA per quarter in the study period from 2013 to 2021.
**Table S6:** Total number of analysed urine samples for SCRAs per quartal in the study period from 2013 to 2021. Number of urine samples positive for at least one SCRA and urine samples positive for each individual SCRA.
**Table S7:** Percentage of urine samples positive for each individual SCRA per quartal in the study period from 2013 to 2021.
**Table S8:** Example data for two intoxication cases used for the calculation of the risk score (*G*_*2*_ und *G*_*3*_), including recorded symptoms, quantified and confirmed SCRAs and other drugs of abuse, as well as the evaluation regarding the Poison Severity Score and Toxicological Significance Score;

## Data Availability

Data are available from the authors on reasonable request.
