# Supplementary material for: Development of a novel risk score reflecting the relative harm potential of synthetic cannabinoids based on prevalence estimates, well‐documented intoxication cases and basic pharmacological data
Source: Addiction. 2025 Dec 21;121(4):870–82. doi: 10.1111/add.70268 (PMC12980303; doi:10.1111/add.70268)
Supplement: Supplementary file 1 — Figure S1: Percentage of the respective SCRAs included in this study in herbal incense samples, serum/blood and urine samples analysed in the Institute of Forensic Medicine Freiburg in the study period 2013 to 2021. Figure S2: Concentration‐displacement curves at the human CB1 receptor derived from the competitive [3H]CP,55940 mediated in vitro receptor affinity assay upon the concentration‐dependent stimulation with the twelve test compounds and two reference compounds. Data given as mean receptor affinity ± SEM (n = 3 or higher). Figure S3: Concentration dependent interaction of [35S]‐GTPγS with the human CB1 upon stimulation with the twelve test compounds. The data is depicted as mean receptor activation ± SEM (n = 3), normalised to the Emax of CP‐55940 (100 %). Table S1: Materials. Table S2: Number of total and individual SCRA intoxications per quarter in the study period from 2013 to 2021. Table S3: Percentage of individual SCRA intoxications in the respective quarter in the study period from 2013 to 2021. Table S4: Total number of analyzed serum/blood samples for SCRAs per quarter in the study period from 2013 to 2021. Number of serum/blood samples positive for at least one SCRA and serum/blood samples positive for each individual SCRA. Table S5: Percentage of serum/blood samples positive for each individual SCRA per quarter in the study period from 2013 to 2021. Table S6: Total number of analysed urine samples for SCRAs per quartal in the study period from 2013 to 2021. Number of urine samples positive for at least one SCRA and urine samples positive for each individual SCRA. Table S7: Percentage of urine samples positive for each individual SCRA per quartal in the study period from 2013 to 2021. Table S8: Example data for two intoxication cases used for the calculation of the risk score (G 2 und G 3 ), including recorded symptoms, quantified and confirmed SCRAs and other drugs of abuse, as well as the evaluation regarding the Poison Severity Score and Toxico [file ADD-121-870-s001.docx]

**Supporting Information**

**Development of a novel risk score reflecting the relative harm potential of synthetic cannabinoids, based on prevalence estimates, well-documented intoxication cases and basic pharmacological data**

Michaela J. Sommer^1,2,3^*, Katharina Elisabeth Grafinger^1,3,4^*, Maren Hermanns‑Clausen^3,6^, Volker Auwärter^1,3^

^1^Institute of Forensic Medicine, Forensic Toxicology, Medical Center – University of Freiburg, Freiburg, Germany

^2^Hermann Staudinger Graduate School, University of Freiburg, Freiburg, Germany

^3^Faculty of Medicine, University of Freiburg, Freiburg, Germany

^4^Institute of Forensic Medicine, Forensic Toxicology and Chemistry, University of Bern, Switzerland

^6^Poisons Information Center, Department of General Pediatrics, Adolescent Medicine and Neonatology, Center for Pediatrics, Medical Center – University of Freiburg, Freiburg, Germany

*these authors contributed equally to this work

**Sample preparation and LC-MS/MS analysis**

**Serum/blood samples**

The analysis of serum/blood samples was performed according to a validated method by Angerer et al.^1^ using a seven-point calibration curve (0.1-2 ng/mL). Samples were extracted in a two-step extraction protocol. In short to 1 mL of serum/blood, 10 μL of internal standard (c = 25 ng/mL) and 0.5 mL carbonate buffer (pH 10) were added, followed by the first extraction using 1.5 mL hexane/ethyl acetate (99/1, vv). This solution was gently mixed for 5 min, followed by centrifugation at 2860 g for 20 min. 1 mL of supernatant were transferred to a new vial and extracted with1.5 mL *n*-hexane/ethyl acetate (80/20, vv), followed by gently mixing for 5 min and centrifugation at 2860 g for 20 min. 1 mL of each supernatant was transferred to one new autosampler vial and evaporated to dryness using N_2_ at 40°C, followed by reconstitution in 100 μL of mobile phase.

Serum/blood samples were analysed on a Shimadzu Prominence HPLC system (Duisburg, Germany) coupled to a AB Sciex QTrap 4000 triple quadrupole liner ion trap mass spectrometer (Darmstadt, Germany) with a Kinetex C18, 100 Å (100 x 2.1 mm, 2.6 μm) column. The mobile phase consisted of water with 1% acetonitrile, 2 mmol/L ammonium formate and 0.1% formic acid (A) and acetonitrile with 2 mmol/L ammonium formate and 0.1% formic acid (B). The following elution gradient was applied: 20% B held for 1 minute, increased to 60% over 1.5 minutes, further increased to 65% over 1.5 min and held constant for 1 minutes, and further increased to 99% B over 3 minutes and kept for 2 minutes. The starting gradient was re-established rapidly in 0.2 minutes (20% B) and held for 1.8 minutes, resulting in a total runtime of 12 minutes.

**Urine samples**

Urine samples were analysed according to previously published and validated method by Franz et al.^2^. For sample preparation 0.5 mL of phosphate buffer (pH 6) and 30 μL of β-glucuronidase were added to 0.5 mL of urine. Incubation was for 60 min at 45°C. This was followed by protein precipitation using 1.5 mL acetonitrile containing the internal standard (c = 2 ng/mL) and 0.56 mL 10 M ammonium formate solution, followed by shaking and centrifugation. 1 mL of the organic phase was transferred into an autosampler vial and evaporated to dryness under N_2_. The samples were reconstituted in 200 μL mobile phase.

Urine sample analysis was performed on a Dionex UltiMate® 3000RS UHPLC (Thermo Scientific, Dreieich, Germany) coupled to an API 5000^TM^ triple quadrupole instrument with a TurboIonSpray® interface (Sciex, Darmstadt, Germany). Separation was performed on a Luna C18 100 Å (150 x 2 mm, 5 μm) column with the mobile phase consisting of 0.2% HCOOH and 2 mmol/L NH^4^+HCOO^−^ in water (A) and acetonitrile (B). Following gradient was applied: starting condition of mobile phase B was 50%, linearly increased to 60% within 3.5 min, further increased to 70% within 2 min, further increased to 75% within 1 min, further increased to 80% within 1 min, further increased to 90% in 1.5 min, held for 2.5 min, decreased to starting conditions of 50% in 0.5 min and held for 3.0 min for re-equilibration.

**
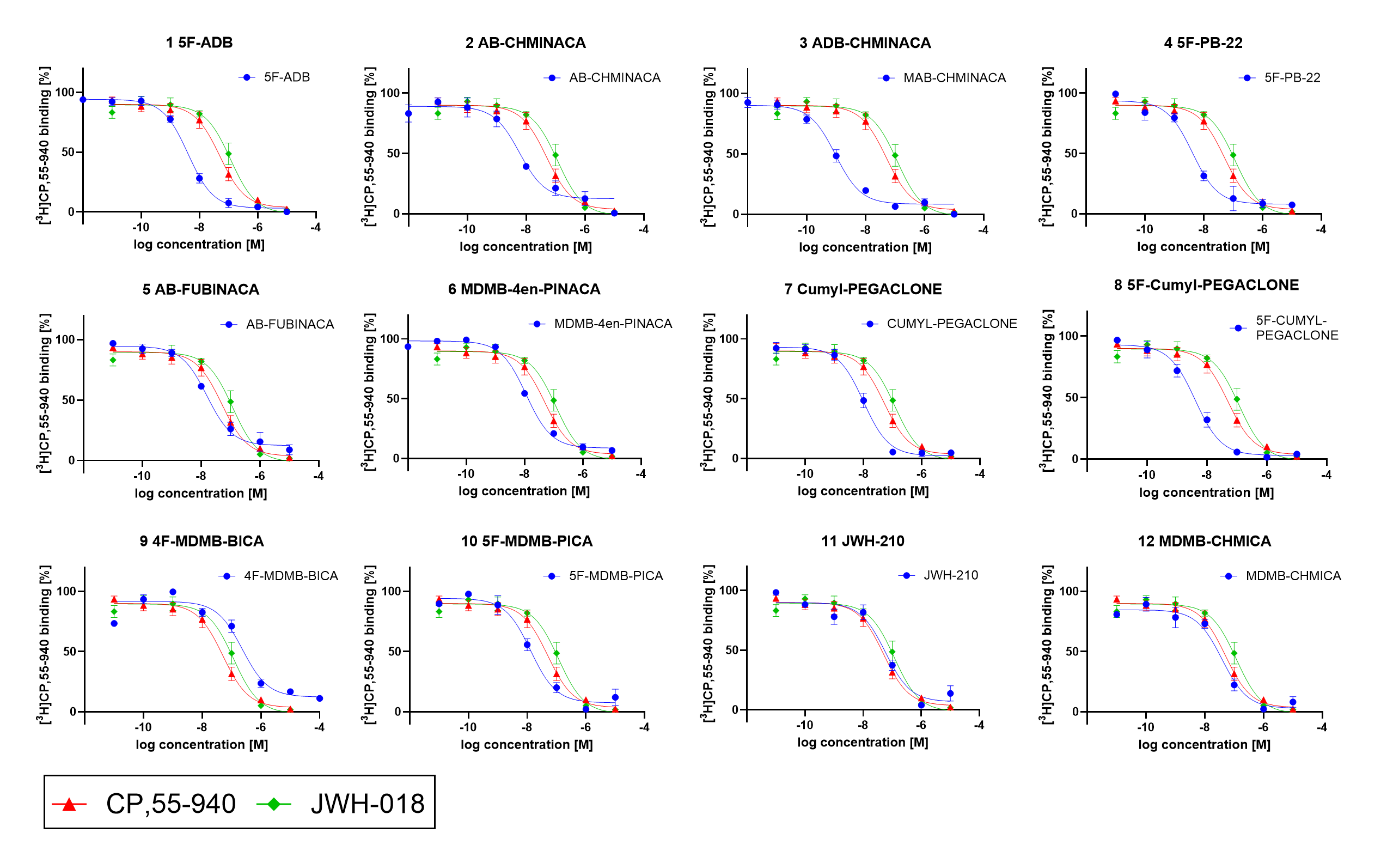
**

**SI Figure 1:** Concentration-displacement curves at the human CB_1_ receptor derived from the competitive [^3^H]CP,55-940 mediated *in vitro* receptor affinity assay upon the concentration-dependent stimulation with the twelve test compounds and two reference compounds. Data given as mean receptor affinity ± SEM (n=3 or higher).


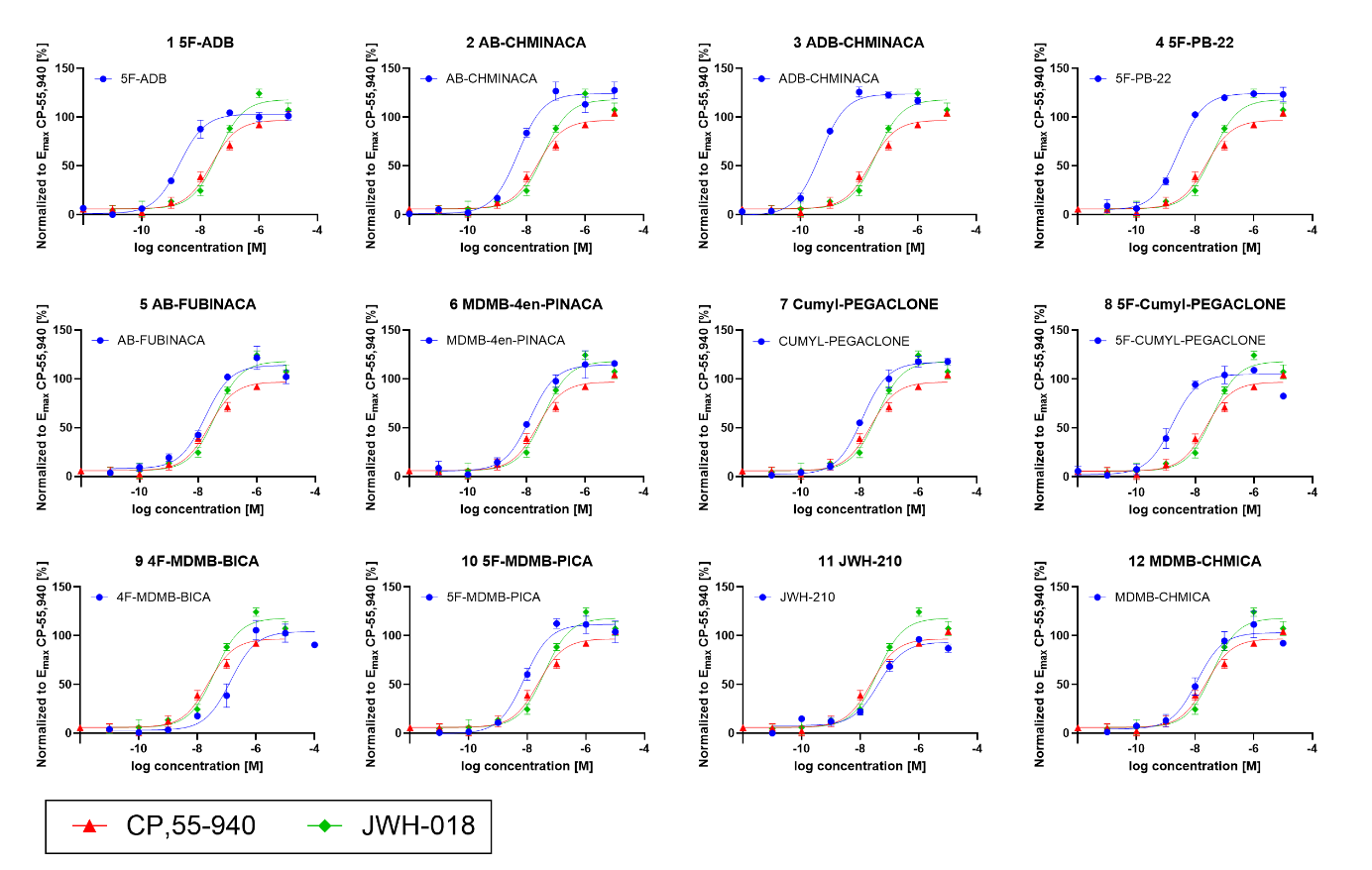
**SI Figure 2:** Concentration dependent interaction of [^35^S]-GTPγS with the human CB1 upon stimulation with the twelve test compounds. The data is depicted as mean receptor activation ± SEM (n=3), normalized to the E_max_ of CP-55,940 (100 %).

**
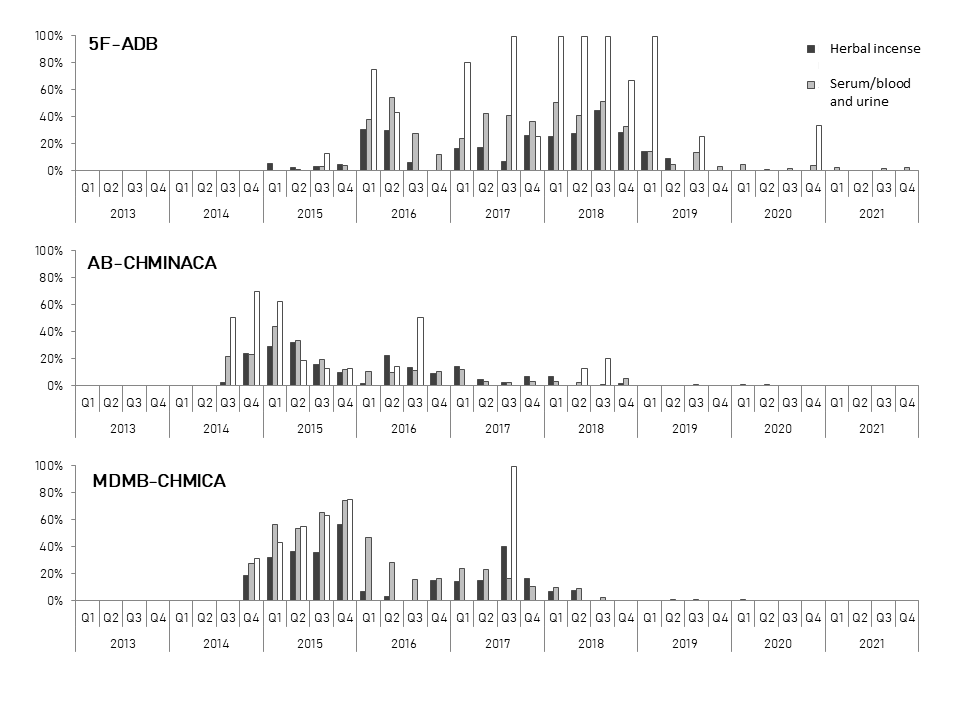
**

**SI Figure 3: Percentage of the respective SCRAs included in this study in herbal incense samples, serum/blood and urine samples analysed in the Institute of Forensic Medicine Freiburg in the study period 2013 to 2021.**

**SI Figure 3 continued**

**
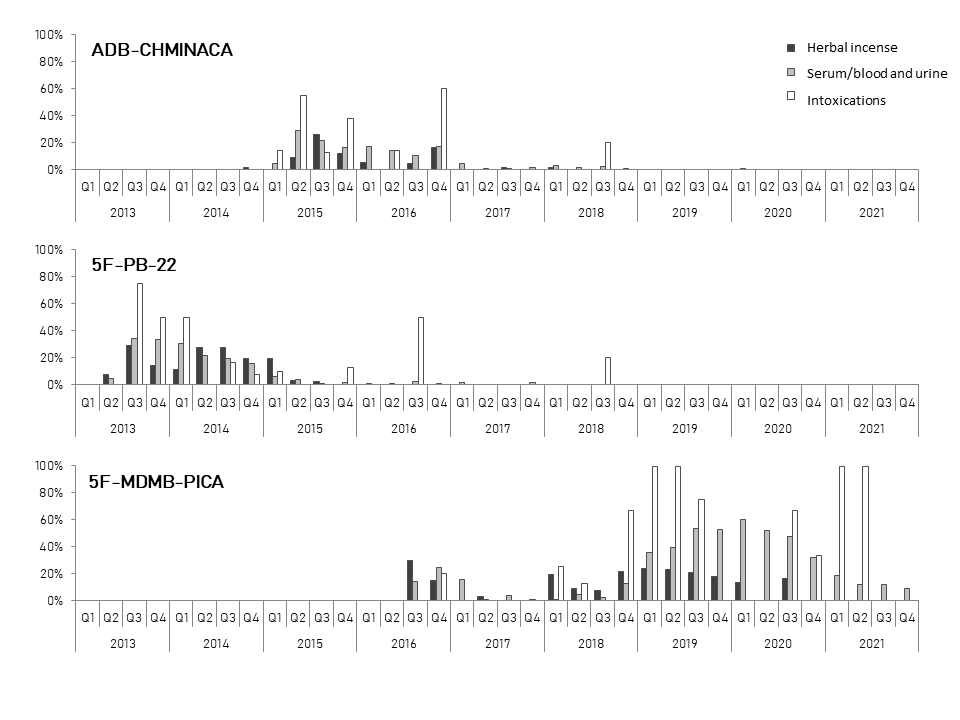

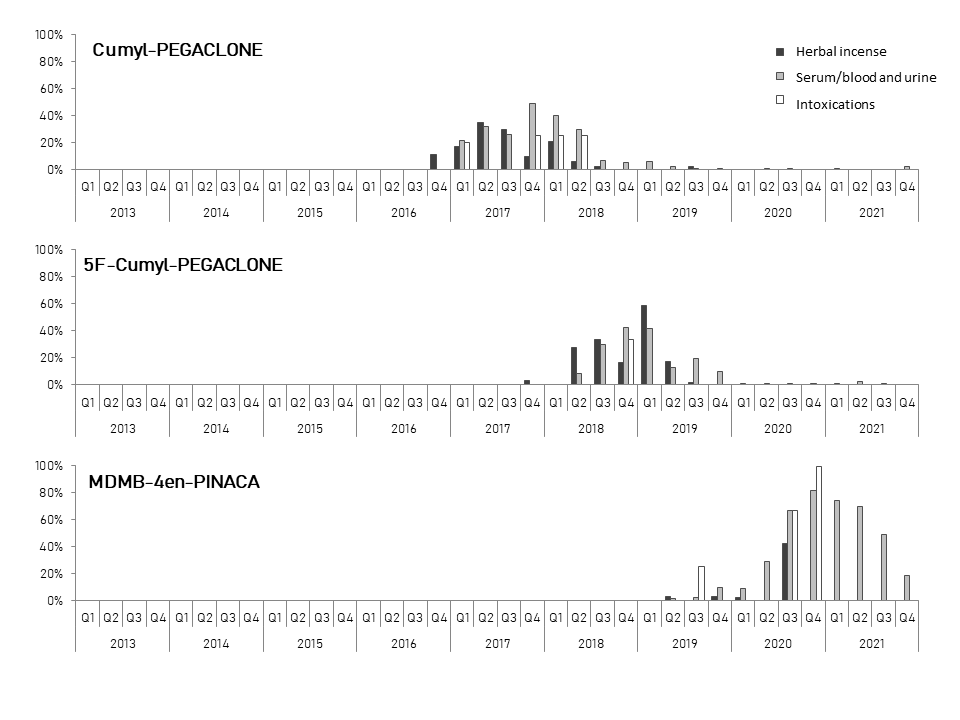
**

**SI Figure 3 continued**

**
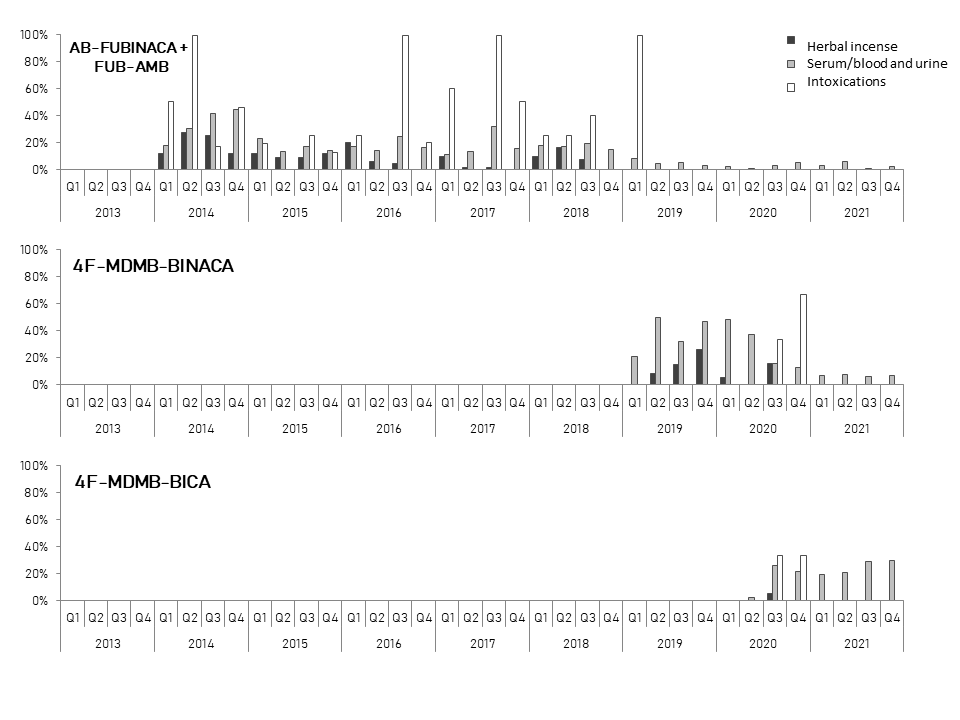

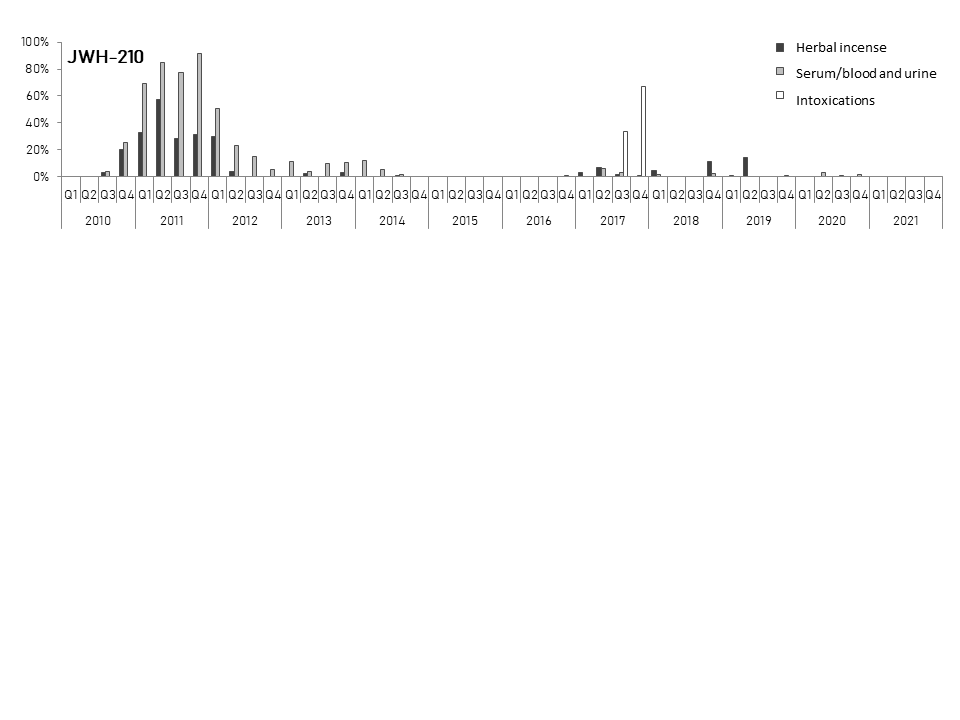
**

**SI Table 1: Materials**

| **Compound** | **Vendor** | **Location of Vendor** |
| --- | --- | --- |
| Absolute ethanol | Sigma Aldrich | Steinheim, Germany |
| MgCl₂ | Sigma Aldrich | Steinheim, Germany |
| TRIS HCl (tris(hydroxymethyl)aminomethane hydrochloride) | Sigma Aldrich | Steinheim, Germany |
| Bovine serum albumin (BSA) | Sigma Aldrich | Steinheim, Germany |
| EGTA (ethylene glycol-bis(β-aminoethyl ether)-N,N,N′,N′-tetraacetic acid) | Sigma Aldrich | Steinheim, Germany |
| GDP (guanosine-5’-disphosphate sodium salt) | Sigma Aldrich | Steinheim, Germany |
| GTPγS (guanosine-5’-(γ-thio)triphosphate tetralithium salt) | Sigma Aldrich | Steinheim, Germany |
| DMSO (dimethyl sulfoxide) | Sigma Aldrich | Steinheim, Germany |
| Sodium chloride | Carl Roth | Karlsruhe, Germany |
| Sodium hydroxide  (≥ 99%, p.a., pellets) | Carl Roth | Karlsruhe, Germany |
| MultiScreen™ filter plates (1.2 μm) | Merck | Darmstadt, Germany |
| Sodium carbonate | Merck | Darmstadt, Germany |
| Isopropanol (Prepsolv®) | Merck | Darmstadt, Germany |
| (±)-CP-55,940 | Cayman Chemical Company | Hamburg, Germany |
| JWH-018 | Lipomed | Arlesheim, Switzerland |
| Human CB_1_ membrane preparations (HEK293-EBNA) | Perkin Elmer | Waltham, USA |
| Ultima Gold™ (liquid scintillation cocktail) | Perkin Elmer | Waltham, USA |
| [³H]CP-55,940 (149 Ci/mmol) | Perkin Elmer | Waltham, USA |
| [³⁵S]-GTPγS (1250 Ci/mmol) | Perkin Elmer | Waltham, USA |
| Deionized water | ELGA (Medica® Pro deionizer) | Celle, Germany |
| 5F-ADB | Cayman Chemicals | Tallinn, Estonia |
| AB-CHMINICA | Cayman Chemicals | Tallinn, Estonia |
| MDMB-CHMICA | Cayman Chemicals | Tallinn, Estonia |
| 5F-PB-22 | Cayman Chemicals | Tallinn, Estonia |
| MAB-CHMINACA | Cayman Chemicals | Tallinn, Estonia |
| AB-FUBINACA | Cayman Chemicals | – |
| 5F-MDMB-PICA | Forensic Institute Zurich | Zurich, Switzerland |
| JWH-210 | Forensic Institute Zurich | Zurich, Switzerland |
| 4F-MDMB-BICA | Forensic Institute Zurich | Zurich, Switzerland |
| MDMB-4en-PINACA | Slovenian National Forensic Laboratory | Slovenia |
| Cumyl-PEGACLONE | Research group of Dr. Samuel Banister, University of Sydney | Sydney, Australia |
| 5F-Cumyl-PEGACLONE | Research group of Dr. Samuel Banister, University of Sydney | Sydney, Australia |

**SI Table 2:** Number of total and individual SCRA intoxications per quarter in the study period from 2013-2021

**SI Table 3:** Percentage of individual SCRA intoxications in the respective quarter in the study period from 2013-2021

**SI Table 4:** Total number of analysed serum/blood samples for SCRAs per quarter in the study period from 2013-2021. Number of serum/blood samples positive for at least one SCRA and serum/blood samples positive for each individual SCRA.

**SI Table 5:** Percentage of serum/blood samples positive for each individual SCRA per quarter in the study period from 2013-2021.

**SI Table 6:** Total number of analysed urine samples for SCRAs per quartal in the study period from 2013-2021. Number of urine samples positive for at least one SCRA and urine samples positive for each individual SCRA.

**SI Table 7:** Percentage of urine samples positive for each individual SCRA per quartal in the study period from 2013-2021

.

SI Table 8: Example data for two intoxication cases used for the calculation of the risk score (*G_2_* und *G_3_*), including recorded symptoms, quantified and confirmed SCRAs and other drugs of abuse, as well as the evaluation regarding the Poison Severity Score and Toxicological Significance Score;

| **Case** | | **sample type** | | **Concentration of evaluated SCRA** | **Recorded symptoms** | | **Other confirmed SCRAs** | **Other confirmed drugs of abuse** | | **PSS** | | **TSS** |
| --- | --- | --- | --- | --- | --- | --- | --- | --- | --- | --- | --- | --- |
| **AB-CHMINACA** | | | | | | | | | | | | |
| A | Serum | | 9.5 ng/mL | | bradycardia (35 bpm/min), seizure, hypokalaemia |  | | | 3-MMC approximately 320 ng/mL | | 3 | 2 |
|  | Urine | | positive | |  |  |  |  | 3-MMC | |  |  |
| B | Serum | | ca. 27 ng/mL | | Tachycardia, nystagmus, disorientation, anxiety,  headaches | 5F-PB-22 ca. 1.5 ng/mL; 5F-AKB-48 < 0.1 ng/mL; THJ-018 < 0.1 ng/mL; THJ-2201 < 0.1 ng/mL | | |  | | 2 | 3 |
|  | Urine | | positive | |  | 5F-AKB-48; PB-22-5F; THJ-018; THJ-2201; FUB-AMB/AB-FUBINACA hint | | |  |  |  |  |

**References**

1. Angerer V, Süssenbach F, Hirschinger N, Auwärter V. Validated LC-MS/MS method for qualitative and quantitative analysis of 75 synthetic cannabinoids in serum. The International Association of Forensic Science 2015; 2015; Florence, Italy.

2. Franz F, Angerer V, Jechle H, et al. Immunoassay screening in urine for synthetic cannabinoids - an evaluation of the diagnostic efficiency. *Clin Chem Lab Med.* 2017;55(9):1375-1384.
